# Author Correction: A computational method for detection of ligand-binding proteins from dose range thermal proteome profiles

**DOI:** 10.1038/s41467-021-27561-5

**Published:** 2021-12-08

**Authors:** Nils Kurzawa, Isabelle Becher, Sindhuja Sridharan, Holger Franken, André Mateus, Simon Anders, Marcus Bantscheff, Wolfgang Huber, Mikhail M. Savitski

**Affiliations:** 1 European Molecular Biology Laboratory, Genome Biology Unit, Meyerhofstrasse 1, Heidelberg, 69117 Germany; 2 Faculty of Biosciences, Heidelberg University, Heidelberg, 69120 Germany; 3 Cellzome GmbH, GlaxoSmithKline, Meyerhofstrasse 1, Heidelberg, 69117 Germany; 4 Center for Molecular Biology of Heidelberg University (ZMBH), Im Neuenheimer Feld 282, Heidelberg, 69120 Germany

**Keywords:** Proteomics, Bioinformatics, Proteome informatics, Statistical methods

Correction to: *Nature Communications* 10.1038/s41467-020-19529-8, published online 13 November 2020.

The original version of this Article contained an error in Eq. (5) and the corresponding notation, and incorrectly read:

“Using the assumption that the residuals follow a normal distribution, Bayes' theorem and the scaled inverse *χ*^2^ prior, it can be shown [20] that the expected value of the posterior of ${\tilde{s}}_{i}^{2}$ is

5 $${\tilde{s}}_{i}^{2}=\frac{{d}_{0}{s}_{0}^{2}+{d}_{2}{s}_{i}^{2}}{{d}_{0}+{d}_{g}}.$$

Here, the hyperparameters ${s}_{0}^{2}$ and ${d}_{0}$ are estimated by fitting a scaled *F*-distribution with ${s}^{2} \sim {s}_{0}^{2}{F}_{d,{d}_{0}}$”.

The correct form of Eq. (5) and the corresponding notation is:

“Using the assumption that the residuals follow a normal distribution, Bayes' theorem and the scaled inverse *χ*^2^ prior, it can be shown [20] that the expected value of the posterior of ${\sigma }_{i}^{2}$ given ${s}_{i}^{2}$ is

5 $${\tilde{s}}_{i}^{2}=\frac{{d}_{0}{s}_{0}^{2}+{d}_{2}{s}_{i}^{2}}{{d}_{0}+{d}_{2}}.$$

Here, the hyperparameters ${s}_{0}^{2}$ and ${d}_{0}$ are estimated by fitting a scaled *F*-distribution with ${s}_{i}^{2} \sim {s}_{0}^{2}{F}_{{d}_{2},{d}_{0}}$”.

In addition, the original version of this Article contained an error in Fig. 1a (step 2), in which the letters of the word “temperature” were scrambled in the gray-white gradient bar.

This has been corrected in the PDF and HTML versions of the Article.

