# Supplementary material for: A computational method for detection of ligand-binding proteins from dose range thermal proteome profiles
Source: Nat Commun. 2020 Nov 13;11:5783. doi: 10.1038/s41467-020-19529-8 (PMC7666118; doi:10.1038/s41467-020-19529-8)
Supplement: Supplementary file 1 — Supplementary Information [file 41467_2020_19529_MOESM1_ESM.pdf]

Supplementary Information: A computational method for detection of ligand-binding proteins from dose range thermal proteome profiles

Kurzawa*, Becher* *et al.*

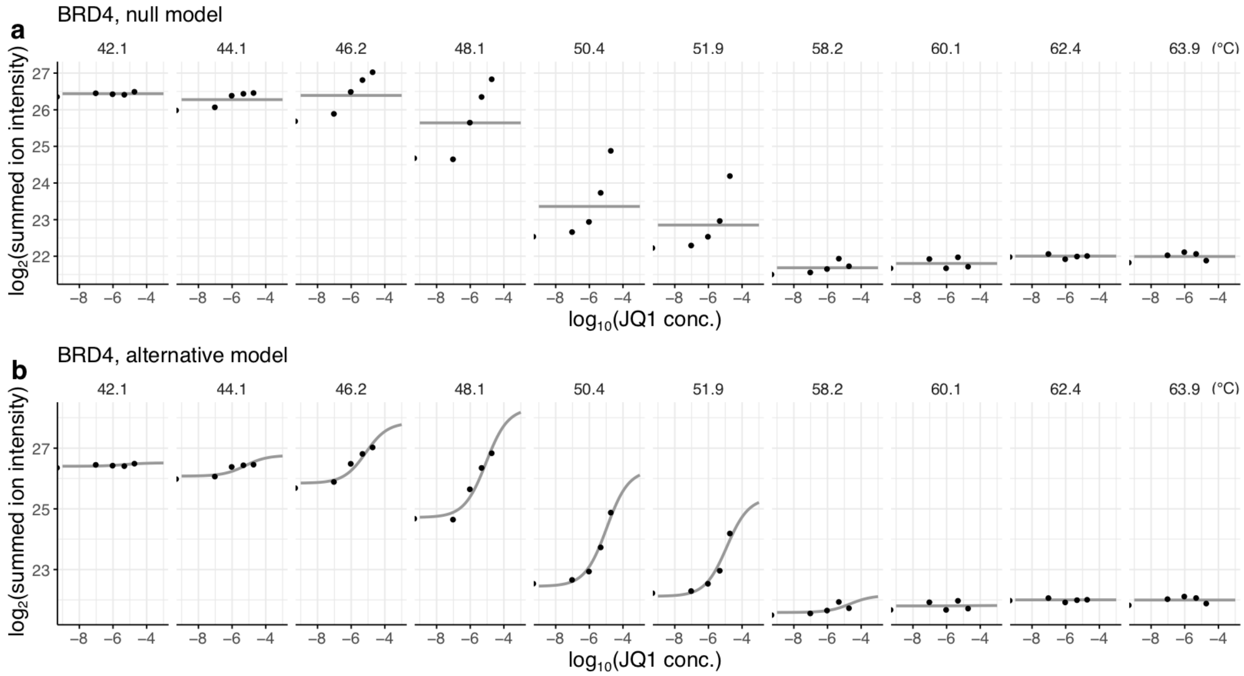

Supplementary Figure 1: **Example of null and alternative model fits to the thermal profile of BRD4 measured in THP1 lysate in the presence of different concentrations of its inhibitor JQ1.**

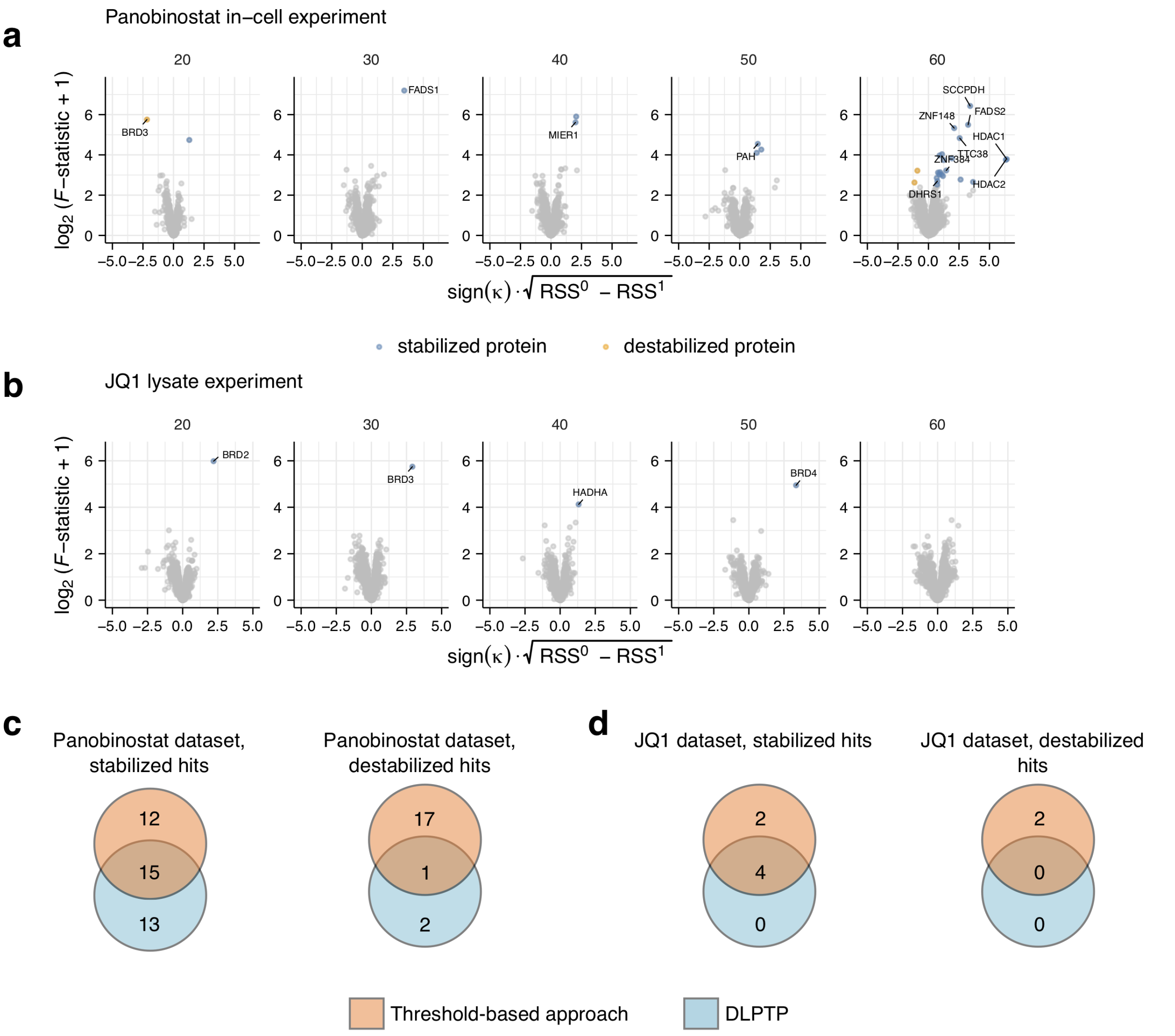

Supplementary Figure 2: **Comparison of 2D-TPP analysis results obtained by DLPTP and the threshold-based approach.** a) Volcano plot for the 2D-TPP experiment of panobinostat acquired in HepG2 cells of proteins grouped by similar number of observations (p), e.g., 30 corresponds to $24 < p < 35$. b) Same as a) for the 2D-TPP experiment of JQ1 in THP1 lysate. c) Venn diagrams comparing hits found in the panobinostat dataset between DLPTP and the threshold-based approach. d) same as c) for the JQ1 dataset. RSS: residual sum of squares; $\mathrm{sign}(\kappa)\sqrt{\mathrm{RSS}^0 - \mathrm{RSS}^1}$: measure of effect size—how much more variance is explained by the alternative model compared to the null—and direction, i.e., positive sign for stabilized proteins, negative for destabilized ones; $\log_2(F\text{-statistic} + 1)$: the transformation is used for visualization purposes only, the addition of 1 guarantees that logarithm-transformed values remain bounded as F approaches 0.

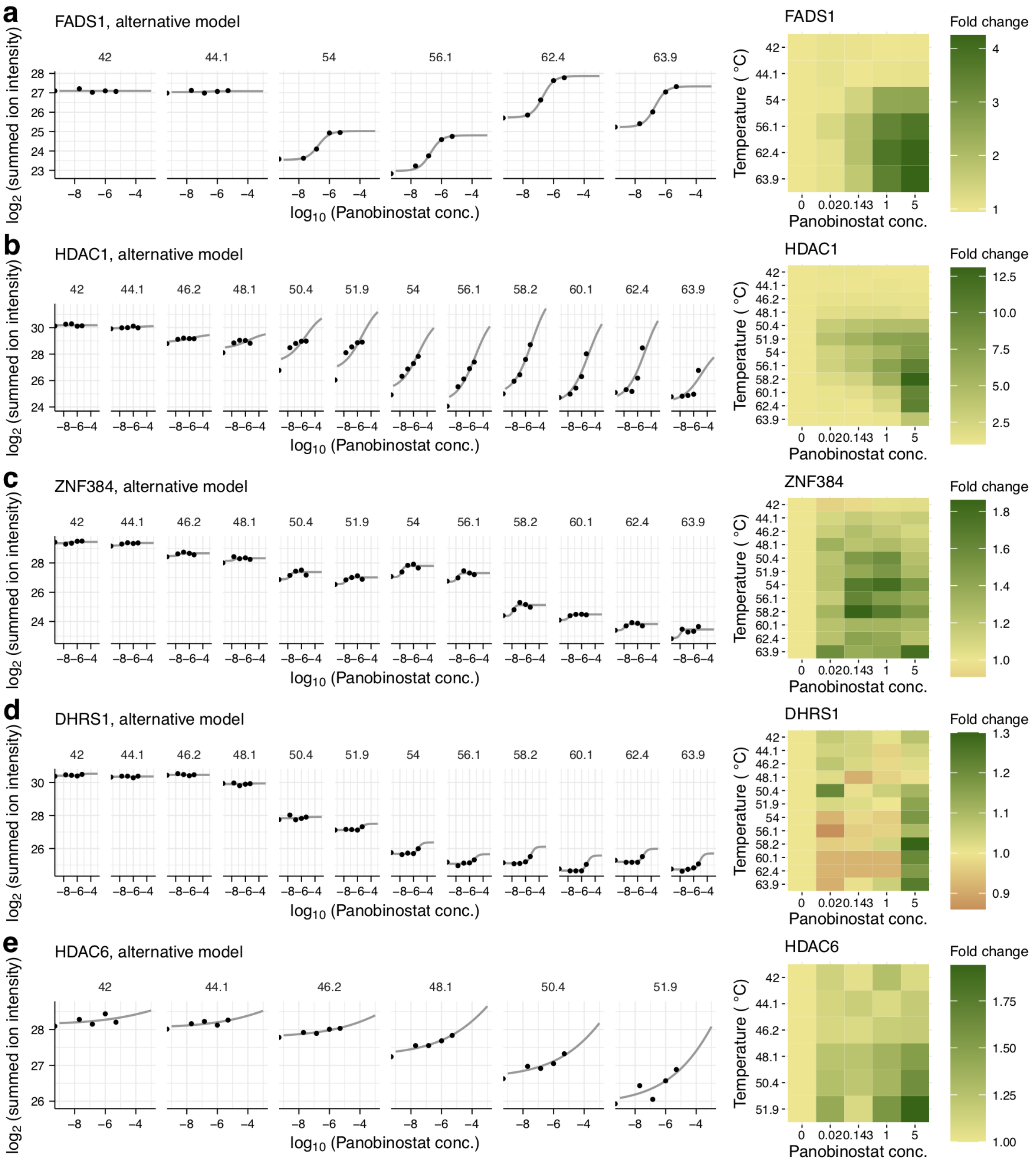

Supplementary Figure 3: **Example profiles of proteins with different *F*-statistics obtained by DLPTP.** a) Acyl-CoA (8-3)-desaturase (FADS1), b) Histone deacetylase 1 (HDAC1), c) Zinc finger protein 384 (ZNF384), d) Dehydrogenase/reductase SDR family member 1 and e) Histone deacetylase 6 (HDAC6).

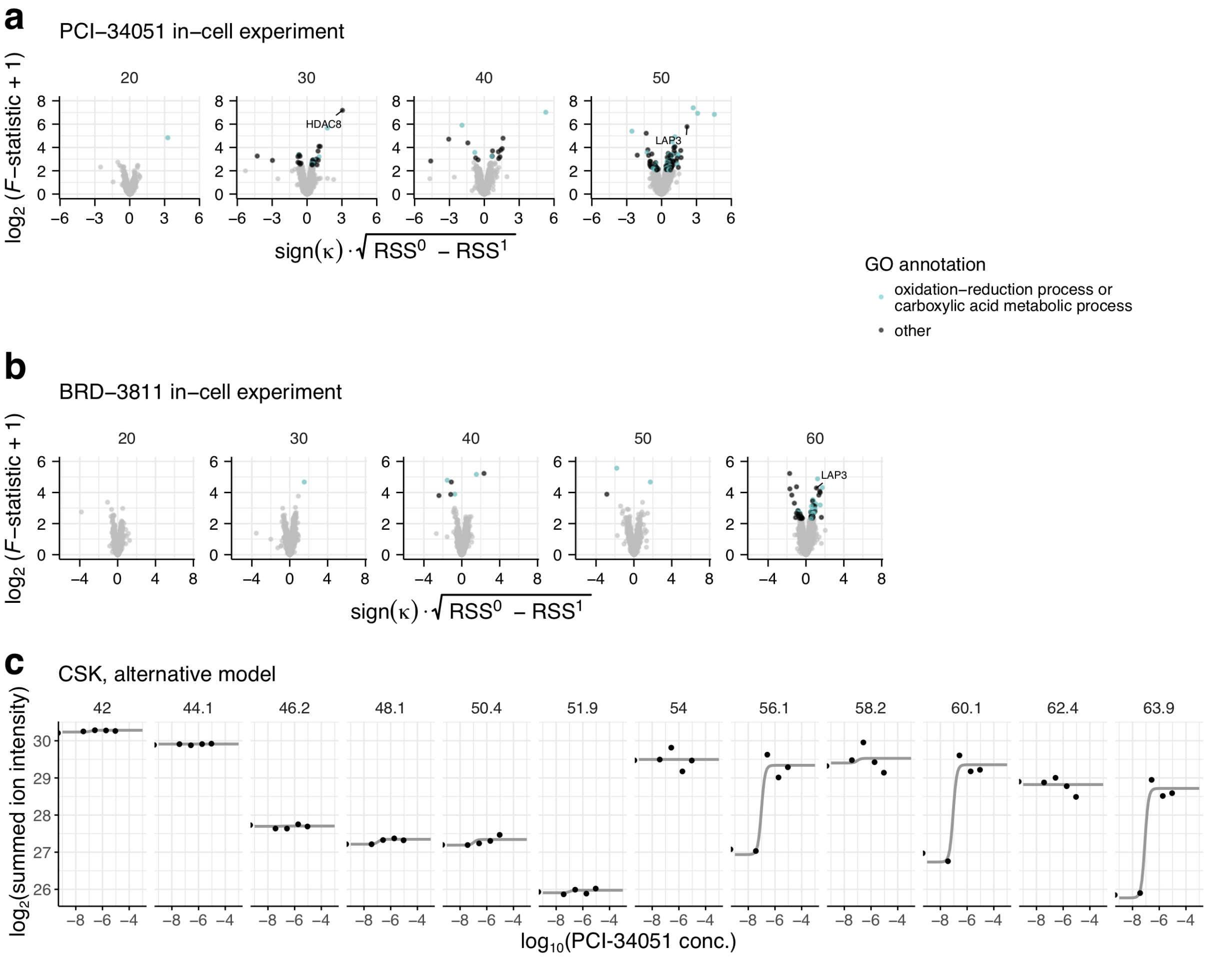

Supplementary Figure 4: **Volcano plots grouped by proteins with similar number of observations for the PCI-34051 and BRD-3811 datasets and example of thermal profile of CSK affected by carry-over.** a) Volcano plot for the 2D-TPP experiment of PCI-34051 acquired in HL-60 cells b) Same as a) for the 2D-TPP experiment of BRD-3811 in HL-60 cells. c) 2D thermal profile of Casein kinase (CSK) obtained in the dataset of PCI-34051 profiled in HL-60 cells. Axes in a) and b) are outlined in Supplementary Figure 2.

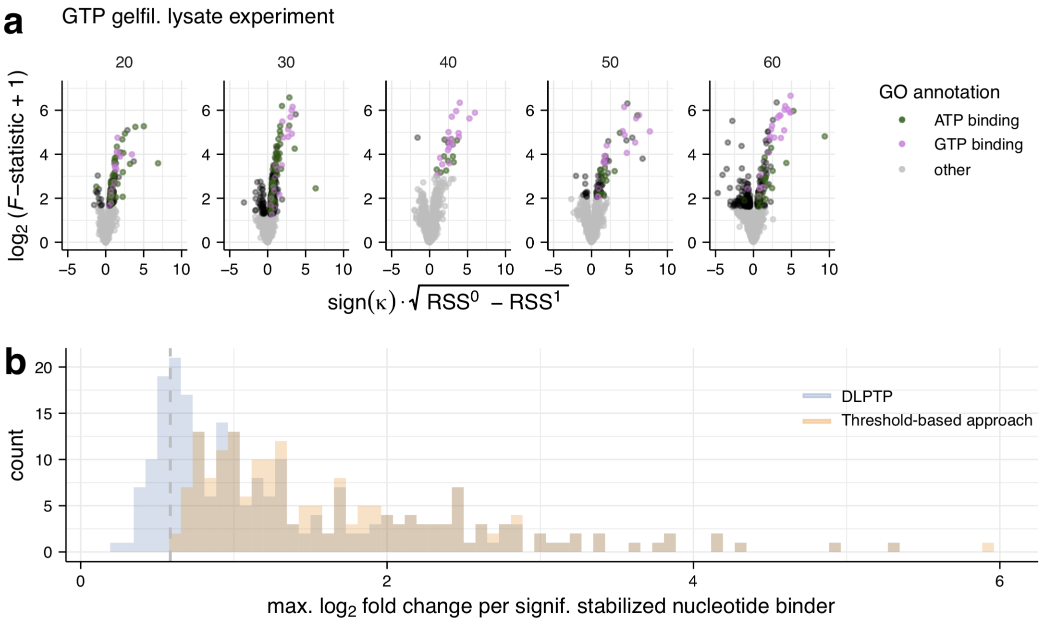

Supplementary Figure 5: **Volcano plots grouped by proteins with similar number of observations for the GTP dataset and histogram of maximal fold changes of significantly recovered nucleotide binders.** a) Volcano plot for the analysis of the GTP gel-filtered lysate dataset grouped by protein with similar number of observations, e.g., 30 corresponds to $24 < p < 35$. b) Histogram of maximal $\log_2$ stabilization fold changes of nucleotide-binders either recovered by the threshold-based approach or by DLPTP. The gray dashed line indicates a fold change of $\log_2(1.5)$.
