# Supplementary material for: A computational method for detection of ligand-binding proteins from dose range thermal proteome profiles
Source: Nat Commun. 2020 Nov 13;11:5783. doi: 10.1038/s41467-020-19529-8 (PMC7666118; doi:10.1038/s41467-020-19529-8)
Supplement: Supplementary file 3 — Description of Additional Supplementary Files [file 41467_2020_19529_MOESM3_ESM.docx]

File Name: Kurzawa_et_al_supplementary_information.pdf

Description: Supplementary Figures

File Name: Supplementary_Data_1.xlsx
Description: Panobinostat 2D-TPP HepG2 cell and JQ1 2D-TPP THP1 lysate dataset and analysis results

File Name: Supplementary_Data_2.xlsx
Description: PCI-34051 and BRD-3811 2D-TPP HL60 cell dataset and analysis results

File Name: Supplementary_Data_3.xlsx
Description: GTP 2D-TPP Jurkat gel fil. lysate dataset and analysis results
