# Supplementary material for: A computational method for detection of ligand-binding proteins from dose range thermal proteome profiles
Source: Nat Commun. 2020 Nov 13;11:5783. doi: 10.1038/s41467-020-19529-8 (PMC7666118; doi:10.1038/s41467-020-19529-8)
Supplement: Supplementary file 7 — Reporting Summary [file 41467_2020_19529_MOESM7_ESM.pdf]

nature research

Corresponding author(s): Mikhail Savitski

Last updated by author(s): Oct 13, 2020

Reporting Summary

Nature Research wishes to improve the reproducibility of the work that we publish. This form provides structure for consistency and transparency in reporting. For further information on Nature Research policies, see Authors & Referees and the Editorial Policy Checklist .

Statistics

For all statistical analyses, confirm that the following items are present in the figure legend, table legend, main text, or Methods section.

n/a	Confirmed	
☐	☒	The exact sample size (*n*) for each experimental group/condition, given as a discrete number and unit of measurement
☒	☐	A statement on whether measurements were taken from distinct samples or whether the same sample was measured repeatedly
☒	☐	The statistical test(s) used AND whether they are one- or two-sided<br>*Only common tests should be described solely by name; describe more complex techniques in the Methods section.*
☒	☐	A description of all covariates tested
☒	☐	A description of any assumptions or corrections, such as tests of normality and adjustment for multiple comparisons
☒	☐	A full description of the statistical parameters including central tendency (e.g. means) or other basic estimates (e.g. regression coefficient) AND variation (e.g. standard deviation) or associated estimates of uncertainty (e.g. confidence intervals)
☐	☒	For null hypothesis testing, the test statistic (e.g. *F*, *t*, *r*) with confidence intervals, effect sizes, degrees of freedom and *P* value noted<br>*Give P values as exact values whenever suitable.*
☒	☐	For Bayesian analysis, information on the choice of priors and Markov chain Monte Carlo settings
☒	☐	For hierarchical and complex designs, identification of the appropriate level for tests and full reporting of outcomes
☐	☒	Estimates of effect sizes (e.g. Cohen's *d*, Pearson's *r*), indicating how they were calculated

Our web collection on statistics for biologists contains articles on many of the points above.

Software and code

Policy information about availability of computer code

Data collection	Proteomics database search was performed using Mascot version 2.5.1 (Matrix Science) and protein quantification was done using isobarQuant V1.10 (https://github.com/protcode/isob/archive/1.10.zip)
Data analysis	All data analysis was perfomed in R version 4.0 and package version 1.5.11 of TPP2D (https://bioconductor.org/packages/devel/bioc/html/TPP2D.html). Custom R code for all central analyses is available from github: https://github.com/nkurzaw/TPP2D_analysis

For manuscripts utilizing custom algorithms or software that are central to the research but not yet described in published literature, software must be made available to editors/reviewers. We strongly encourage code deposition in a community repository (e.g. GitHub). See the Nature Research guidelines for submitting code & software for further information.

Data

Policy information about availability of data

All manuscripts must include a data availability statement. This statement should provide the following information, where applicable:

- Accession codes, unique identifiers, or web links for publicly available datasets
- A list of figures that have associated raw data
- A description of any restrictions on data availability

All acquired mass spectrometry datasets (2D-TPP experiment of GTP treated gel-filtered Jurkat lysate, PCI-34051 and BRD-3811 treated HL-60 cells) were deposited on PRIDE (accession number: PXD016640). Re-analyzed datasets profiling Panobinostat and JQ1 were downloaded from the publishers' websites (DOI: 10.1038/nchembio.2185 and DOI: 10.1016/j.cell.2018.02.030). Uniprot data was downloaded from https://www.uniprot.org/proteomes/UP000005640.

Field-specific reporting

Please select the one below that is the best fit for your research. If you are not sure, read the appropriate sections before making your selection.

- [x] Life sciences
- [ ] Behavioural & social sciences
- [ ] Ecological, evolutionary & environmental sciences

For a reference copy of the document with all sections, see nature.com/documents/nr-reporting-summary-flat.pdf

Life sciences study design

All studies must disclose on these points even when the disclosure is negative.

Sample size	no sample size calculation was performed, sample sizes were chosen based on previous publications performing 2D-TPP experiments
Data exclusions	Data that was excluded comprised 2D-TPP measurements profiling PCI-34051 with noise-level strongly above expected values and protein thermal profiles affected by carry-over in the PCI-34051 and BRD-3811 datasets, a statement on the details of this is present in the methods section
Replication	2D-TPP experiments were performed in single replicates as previously, due to high costs and required instrument time. Novel findings were either validated by literature or independent experiments with orthogonal assays measured in duplicates.
Randomization	No randomization was performed since no human or animal subjects were studied.
Blinding	No blinding was performed since experiments were not particularly susceptible to biasing.

Reporting for specific materials, systems and methods

We require information from authors about some types of materials, experimental systems and methods used in many studies. Here, indicate whether each material, system or method listed is relevant to your study. If you are not sure if a list item applies to your research, read the appropriate section before selecting a response.

Materials & experimental systems

n/a	Involved in the study	
x		Antibodies
	x	Eukaryotic cell lines
x		Palaeontology
x		Animals and other organisms
x		Human research participants
x		Clinical data

Methods

n/a	Involved in the study	
x		ChIP-seq
x		Flow cytometry
x		MRI-based neuroimaging

Eukaryotic cell lines

Policy information about cell lines

Cell line source(s)	HL60 were bought from DSMZ (ACC-3), Jurkat cells were bought from ATCC (TIB-152)
Authentication	Authentication was done using a Promega kit based on polymorphic short tandem repeat profiling
Mycoplasma contamination	Cells used were confirmed negative for mycoplasma contamination
Commonly misidentified lines (See ICLAC register)	To the best of our knowledge no misidentified cell lines has been used
